# Relating business model innovations and innovation cascades: the case of biotechnology

**DOI:** 10.1007/s00191-018-0561-9

**Published:** 2018-03-07

**Authors:** Jorge Niosi, Maureen McKelvey

**Affiliations:** 1 School of Management, UQAM, Université du Québec à Montréal, Montreal, Canada; 2 School of Business, Economics and Law, Department of Economy and Society, Institute of Innovation and Entrepreneurship, University of Gothenburg, Gothenburg, Sweden

**Keywords:** Innovation cascades, Biotechnology, Science-based industries, Technological trajectories, Path creation, Business models, Business model innovation, L26 Entrepreneurship, O31 Innovation and Invention, 032 Management of Technological Innovation and R&D 033 Technological Change: Choices and Consequences, Diffusion

## Abstract

This article conceptualizes innovation as a process, where the scientific and industrial application of technological knowledge nurtures new routines and institutions, in order to relate changing business model innovations to innovation cascades. Innovation in science-based, high-tech sectors is changing its tempo, from the evolutionary pace of incremental novelties punctuated by occasional radical novelties, to innovation cascades. These cascades involve a long series of interlinked radical innovations, which can be traced through various scientific and technological indicators like patents and publications. Innovation cascades are relevant to industry, because they make the future less predictable. They are particularly interesting because these changes also enable the testing an abundance of new business models. Innovation cascades have a major impact on the number and sustainability of business models and on strategy. Business model innovations are visible not only in the existing organizations that undergo change, but also new organizational models appear. The case of biotechnology after the 1980s is used to illustrate our conceptualization.

## Introduction

This article conceptualizes innovation as a process, where the scientific and industrial application of technological knowledge nurtures new routines and institutions, and in order to relate changing business model innovations to innovation cascades. Business models are sets of capabilities that determine how a company creates and captures value (Teece 2010). They consist of a value proposition, a value-chain structure and revenue-generation practices. Lane and Maxfield (1996), Bonvillian (2002) and then Lane (2003) introduced the concept of innovation cascade, which highlights underlying aspects of complexity and system dynamics, to analyze the positive feedback relationship between new artefacts, organizations and attributes. Our focus is upon radical impacts of innovation cascades, specifically in the science-based sectors, using the case of biotechnology to illustrate our conceptualization of the impact on business model innovations.

We would argue that the fundamental idea of innovation cascades appears, under another name, in Schumpeter’s *Business Cycles* (1939). In his book, innovation appears in sudden bursts of radical changes, one creating the condition for the next rapid wave. Thus the railroad attracted demand for cheap steel, but railroad companies also introduced organizational innovation, creating the new corporate form. Schumpeter shows a dynamics (not yet a system dynamics as we analyze it today) where technological innovation spurs industrial growth through new types of organizations. In the United States, financial innovation followed, with the proviso that corporate bonds could be converted into preferred stock. A division of labour among top managers became frequent in large corporations. Banks introduced the centralized structure, abandoning the decentralized types of organization that were used before. Also, new forms of credit appeared to provide financial support for the very large investments required by the building of transcontinental railroads. Imitation and constant recombination of new and old technologies and organizational forms amplified the dynamic effect of the cascade. Schumpeter proposed a powerful idea about the dynamics of the innovation process. But his theory which tried to explain business cycles was never popular. Instead, Keynesian theory of business cycles prevailed.

Later on, radical innovation was deemed analogous to “saltation” in biological change, where short periods of rapid structural change interrupted long periods of stasis and incremental change. In the postwar period, the concept of radical innovation appeared in Britain in the works of Gibbons and Littler, (1979), Rothwell (1980), Freeman and Perez (1988) and others. A few years later, numerous authors were discussing the multifarious dynamics between radical organizational and radical technological innovation (Ritala and Hurmelinna-Laukkanen. 2013; Alexander and Van Knippenberg, 2014). Nelson and Winter (1982) developed the general theory of evolutionary economic change. A certain analogy with biological change was also made in their seminal book, including the idea that organizational competencies and routines were needed, as well as selection mechanisms within firms, markets and institutions. Management science pointed out that companies and governments alike avoid rapid technological change that may devalue their assets and cannibalize their products (Christensen and Bower, 1996). Although point-wise radical innovation processes are analyzed, still much analysis has focused upon innovation processes as proceeded in an incremental fashion, one step at a time (Arthur, 2009; Basalla, 1988).

Section 2 presents our conceptualization of the relationships between business model innovation and innovation cascades. A new conceptualization is needed, because innovation in science-based, high-tech sectors is changing its tempo, from the evolutionary pace of incremental novelties punctuated by occasional radical novelties, to a new period characterized here as innovation cascades (Niosi 2016; McKelvey and Orsenigo 2006). These cascades involve a long series of interlinked radical innovations, which can be traced through various scientific and technological indicators like patents and publications. Innovation cascades are relevant to industry, because they make the future less predictable. They are particularly interesting because these changes also enable the testing of an abundance of new business models. Innovation cascades have a major impact on the number and sustainability of business models and on strategy. Business model innovations are visible not only in the existing organizations that undergo change, but also new organizational models appear.

Sections 3 and 4 illustrates through our analysis of the empirical case of biotechnology. Biotechnology is here defined from its technological knowledge and we recognize its application in different industries (McKelvey et al. 2004; McKelvey and Orsenigo 2006) used to illustrate our conceptualization of these processes is the biotechnology industry, which has undergone tremendous changes in recent decades. We argue that in order to understand the science-based industries like biotechnology, one must understand how innovation cascades require both firm capabilities – analyzed through resource-based view and business innovation models – as well as a focus upon the particular role of star scientists, e.g. their intellectual capital and particular role in this type of innovation cascade.

Moreover, as discussed in the concluding Section 5, the policy implications have been barely analyzed as well as offering new lines of research. The innovation policy of cascades is different from the policy behind the nurturing of normal science and technology. These are complex technologies that require major efforts to develop. They require the cooperation of multiple firms, research universities and public laboratories. They need national and international networks of innovators. They need both the use of existing markets and the creation of new ones. They blur some boundaries between industries and technologies.

## A conceptualization of the relationships between business model innovations and innovation cascades^1^

Innovation cascade is a new concept that is fighting for its admission in the corpus of normal innovation literatures. We will below explain how we find it is a useful way of bringing together various aspects of change, and especially for explaining radical changes in science-based industries.

Our conceptualization is that innovation processes need to relate business model innovations to innovation cascades. Hence, the conceptualization must include an understanding of how firms create value, with a particular emphasis on the resource-based theory of the firm.

### Business model innovations

Business model innovation includes both the organizations, the institutions, and the network structures. The concept of business model innovation has been very popular in recent years (Chesbrough 2010; Massa and Tucci 2013). Business models are sets of capabilities that determine how a company creates and captures value (Teece 2010). They consist of a value proposition, a value-chain structure and revenue generation practices. Their clear specification and testing are very important, as millions of dollars are often lost by the implementation of flawed business models (Magretta 2002). Open business models have become fashionable. “Open business models” according to Chesbrough (2007), are those that use knowledge and capabilities stemming from external organizations to create value, such as those created in universities, government laboratories or other firms. Massa and Tucci ((2013:423) provide a broad concept, as “depicting the rationale of how an organization (a firm or other type of organization) creates, delivers and captures value (economic, social, or other forms of value) in relationship with a network of exchange partners.”

From the perspective of evolutionary economics and studies of radical innovation, changes in technological knowledge and in value creation are related to institutional change. North (1991:97) explained how institutions define choice sets to determine transaction and production costs, and he defined institutions as “the humanly devised constraints that structure political, economic and social interaction. They consist of both informal constraints (sanctions, taboos, customs, traditions and codes of conduct) and formal rules (consitutions, laws, property rights). A more complex view of institutions affecting economic behavour can be found in evolutionary economics. Antonelli (2009) argues that within the economics of innovation field, innovation is now viewed as “a complex, path-dependent process characterized by the interdependence and interaction of a variety of heterogeneous agents, able to learn and react creatively with subjective and procedural rationality”. He goes on to argue that studying complexity requires the following assumptions: 1) Heterogeneous actors; 2) Location matters; 3) Local knowledge; 4) Local context of interaction; 5) Creativity; and 6) Systemic interdependence.

Hence, what kind of adaptive organizational processes can we expect under rapid technological change? According to Eisenhardt and Tabrizi (1995), organizational adaptation for fast product innovation is based on two main types of models: the “compression model” using rational planning and reducing the sequence of the organizational change, and the “experiential model”, that takes into consideration a high degree of uncertainty and uses improvisation, flexibility and trial and error. Similarly, Chesbrough (2010) finds that experimentation is a key activity in the design of business models. Demil and Lecock (2010) argue that experimentation cannot be avoided: firms design their business models in a fine-tuning process. In this perspective, Malhotra and Hinings (2015) found, through the analysis of three firms, that evolutionary change is the more frequent situation. Because a business model is a set of business practices that includes a value proposition, a customer base, distribution channels, basic resources and revenue streams, they will change over time. What we find particularly interesting is that business model innovation is an organizational change that substantially alters the activities, resources and revenue stream of the firm. Patents may protect business models, but some of them go unnoticed (Abramowicz 2011). Hence, business model innovation includes technological change, but also organizational and institutional change.

### Innovation cascades and innovation systems: complexity, and multi-stability

Lane and Maxfield (1996), Bonvillian (2002) and then Lane (2003) introduced the concept of innovation cascade. In spite of its intuitive appeal, the concept did not become a rapid success, probably because it had a modest launching pad in working papers and conference proceedings. However, Lane insisted and published several other papers – again in book chapters, conference proceedings and working papers, seldom in journal articles. However, he managed to link innovation cascades to the concepts of complexity and system dynamics, which we feel is most useful for understanding innovation processes. Lane used the term “exaptive bootstrapping” for the positive feedback relationship between new artefacts, organizations and attributes.

“ The resulting dynamics of innovation processes can generate a positive feedback, which works like this: (1) new artefact types are designed to achieve some particular attribution of functionality; (2) organizational transformations are constructed to proliferate the use of tokens of the new type; (3) novel patterns of human interaction emerge around these artefacts in use; (4) new attributions of functionality are generated to describe that the participants in these interactions are obtaining or might obtain from them; (5 = 1) new artefacts are designed to instantiate the new attributed functionality. The innovation cascades that result from this positive feedback dynamic, characterized as they are by the generation of new attributions and the emergence of new patterns of agent interaction, are anything but linear and predictable”. (Lane, 2012)^2^

Antonelli (2008) analyzed the diffusion of innovation to pecuniary externalities, those that take place through the market mechanism. These spillovers are different from technological externalities that take place outside markets. In Antonelli’s work, one can understand how innovation cascades occur within local and sectoral innovation systems. Co-localised firms, and firms that interact with the original innovator within the sector, can more easily appreciate the cost reductions and opportunities involved in the new products and organizational forms, and eventually generate new ones. The radical innovation ceases to be a unique phenomenon and becomes a cascade within the region.

Hence, innovation systems are a relevant concept, in order to understand institutional change and the networks between heterogeneous actors (Nelson 1993; Lundvall 1992). Innovation systems can be national, regional or sectoral, and consist of constituent actors, knowledge, networks and institutions.

As an illustration, one can therefore understand that an innovation cascade involving a series of radical changes can occur within a knowledge domain or single sector as well as across several of them. For example, modern biotechnology based on genetic engineering has affected the scientific field of biology as well as many sectors, ranging from agriculture to health. More recently, genomics has had important effects on the pharmaceutical industry and the health services industry. The mutual externalities between informatics and genomics have produced increasingly fast, reliable and cheaper gene sequencers and bioinformatics software. Likely, the vast majority of innovation cascades take place within sectors; they do not destroy the boundaries of sectoral systems of innovation, but instead reconfigure them in terms of organizations, linkages, artefacts, and attributes of functionality. There does seem to be a concentration in regional systems of innovation. Knowledge likely diffuses first within metropolitan areas, due to mechanisms that were well explained by both Marshall and Jacobs. Thus, there are biotechnology regional innovation systems (Cooke, 2002; Holmén and McKelvey 2005), as there are ICT clusters, aircraft and other regional systems. We thus predict that there are multiple ways in which innovation systems may be linked to innovation cascades.

### Breaking the predictability of technological trajectories: propositions on the where and when of innovation cascades

Due to our evolutionary perspective on innovation and growth, we are interested in quite radical change, which may get started in a multitude of ways, and that many forces must interact for an innovation cascade. The very concept of a technological trajectory (Dosi, 1982) like the concept of scientific paradigm (Kuhn 1962) implies the idea of predictability in normal periods of scientific advancement. Similarly, institutional and organizational trajectories do exist (Zysman, 1994). Indeed, innovation cascades do not obliterate the usefulness of time-honoured ideas about evolutionary innovation or the dichotomy of incremental versus radical innovation. Innovation cascades should be seen as a theoretical construct, which is an addition to the dichotomy incremental/radical innovation, not a rejection of it.

But what is conceptually interesting is that innovation cascades are defined as launching new scientific paradigms (scientific revolutions) and new technological trajectories (Coccia 2012). Lane (2012) identified the first innovation cascade: the arrival of the pocket book in Venice around 1500, which led to the invention of the italic characters, improvements in paper and ink, and rapid diffusion of knowledge through a steep reduction in the cost of books. Hence, authors argue that these cascades break the predictable trajectories of technology and organization (Lane et al., 2009; Berkers & Geels, 2011; Suenaga, 2015; Svetiev, 2011; Winder, 2007), thereby heading developments in new directions. The cascades are emerging properties of previous technological trajectories, yet unpredictable in direction. The scientific knowledge base changes so fast that even those who are supposed to be the most knowledgeable about the technology are often confounded.

Innovation cascades are becoming increasingly abundant for many reasons, and yet they seem concentrated in some geographical spaces and some time frames. At the time of the invention of the modern printing press, the centres of knowledge production and diffusion were a short list of cities such as Florence and Venice in Italy, Amsterdam in the Netherlands, Paris in France, London in Britain and Madrid in Spain.

We are not aware of innovation cascades taking place in developing countries, but occasionally such countries produce a radical innovation. As an illustration, even though developing countries have entered into biotechnology, they are not leading nor has their share increased, although there are regional and sectoral differences (Niosi et al. 2012). Mokyr (1990) suggested that many Chinese innovations (advanced ships, the printing press) were either suppressed or controlled by bureaucratic restraint of the Ming dynasty (1368–1644), and their diffusion was sometimes forbidden by the central government. In Europe, political divisions favoured the diffusion of advanced scientific or technical ideas from one country to others. No autocratic European ruler or the Catholic Church could completely suppress technical and scientific advancement in Europe, thus leaving allowing innovation cascades to emerge.

Thus, before the nineteenth century, innovation cascades were rare events and usually petered out very fast. The reasons are many: First, the institutional environment did not contribute to its adoption, but blocked the diffusion of innovation and the emergence of new radical ones: indexes of forbidden books were numerous and censorship was widespread. Also, universities and private companies did not conduct much research, if any, and there were no public research laboratories to push the cascade further. Radical innovation depended on the individual efforts of remarkable luminaries like Galileo, Da Vinci, or extraordinary artisans such as Aldo Manuzio. At that time, the innovation centres of the world were limited to just a few cities and within them there were few innovating organizations. Also, communication between those centres was slow and costly, and the scientific and technical knowledge of the times was scanty. Innovation came through serendipity, and was not the routine activity of many organizations as it is today.

Present day innovation cascades in high-tech sectors are more frequent than any time in the past. Also, they are anything but predictable. The reasons are many, and six of them include:

1. Before WWII, the number of science-based high-tech sectors was very small. Aeronautics, antibiotics, chemicals and scientific management spread over these new industries as well as older ones.
2. Innovation cascades had many loci, as a growing number of institutions, regional clusters and countries contributed to their development. Up to the mid twentieth century only a handful of countries in Western Europe and North America produced advanced technology. Today, an increasing number of nations have added themselves to the cascades, most of them in Eastern and Southern Asia: Japan, South Korea, India, Taiwan, and Singapore but also, Australia. Thus, in 1980 some eleven countries had registered only 74 patents in the OECD. Half of these patents belonged to US assignees. In 2013, the latest year for which the OECD published data, forty-nine countries had almost 11,000 patents. Three quarters of these patents belonged to American and European Union assignees. But many other countries were also part of the cascade, in Asia and America.
3. In addition, the entry of these new countries was amplified by the arrival of new organizations both in the old and new countries: new research universities, public research organizations, and many more innovative firms. Nature Biotechnology lists the 30 universities with the largest number of biotechnology patents, most of them American universities. The number of academic biotechnology patents has also increased very fast in the United States, particularly after 2012 (Huggett and Paisner, 2014). More countries and institutions conduct biotechnology R&D today than ever before, thus there is more innovation. These 30 universities had obtained 2251 US patents.
4. Generic technologies have impacts in many different sectors. Also, their systemic effects are increasingly global, as these technologies are easily transferred from the original innovating nation to the next, and international research collaboration accelerates the speed of innovation. Many more new ideas are produced, easily and quickly transferred, modified, redeployed and used today than before WWII. Faster and cheaper telecommunication equipment and transportation systems allow novelties to diffuse at high speed. International scientific and technological collaboration contributes to the diffusion and growth of science-based technologies.
5. Many of these new technologies are born in the United States and the Western Europe after WWII. Today they diffuse widely, and such countries as Brazil, China, India, and South Korea contribute to the cascade. The same multiplication of innovative organizations could be found in aerospace, ICT and nanotechnology. The result is the same: more and faster innovation, and quicker diffusion.
6. Modern cascades thus are ultimately born of the rapid development and diffusion of science-based industries after World War II.

## The case of biotechnology

Biotechnology includes a series of scientific disciplines as well as technological knowledge applied in many different sectors. Our definition includes core aspects of the underlying scientific disciplines which can be applied in many sectors (McKelvey et al. 2004; McKelvey and Orsenigo 2006). This article primarily focuses upon biotechnology in pharmaceuticals and biotechnology, but provides some evidence in other sectors like agriculture. In biotechnology, with its ever-growing number of disciplines and discoveries, the number of business models has multiplied in the last 40 years since the development of genetic engineering and the creation of Genentech in 1976. We will traces the development of the disciplines and the emergence of the new business models. So what factors have determined the multiplication of these business models?

### Factors influencing the emergence of new business models in biotechnology

Besides new technologies, the national institutions have also an impact on business models. The differential institutions countries allow or prohibit, foster or discourage can explain national differences in business models. Henc, one aspect is institutional factors, both at the national, regional and sectoral levels of the innovation systems. Casper and Kettler (2001) underlined the key influence of national institutions on business models in biotechnology. They pointed to the fact that the institutions in the United States (numerous research foundations, national health institutes and other public R&D laboratories, the largest venture capital market in the world, and technology transfer offices in all research universities) have nourished the biotech entrepreneurial model, so prominent in that country. Also, with a similar labour market and similar universities as the one found in the United States, the United Kingdom has always been far ahead of any other European country in the biotechnology sector.

As to the main determinants of experimentation with business model innovations in biotechnology, McKelvey (2004) specifies institutional features of the biotechnology sectoral system of innovation. She defines a series of institutional determinants, which in turn stimulate firms to innovate, take risks and try to commercialize new ideas. She argues that the most important institutional variables which affect experimentation with business model innovation are:

- By providing resources and incentives for research, development and innovation
- Using public monies to stimulate the commercialization of new technology, instruments, models, databases, and so forth
- Setting the institutional conditions for new business opportunities (i.e. what is patentable)
- Stimulating reform of regulations and institutions
- Influencing existing demand
- Highlighting new types of economic value (e.g. what customers are willing to pay for)
- Helping to express and form future demand
- Specifying new combinations of goods and services to address health care issues.”

According to McKelvey (2004), the launching of new business models in biotechnology cannot get by without the support of the institutional structure and local and regional market demand in the relevant sectoral system of innovation. She insists on the fact that “Conceptualising biotech firms as specialised suppliers of knowledge also helps us to understand other components of the currently dominant business model within this sector. Many firms do not sell products.” Hence, the ability of the new biotechnology firms to sell products and services can be heavily influenced by institutional factors, when combined together with regional, national and global market demand and available knowledge externalities.

Our own analysis of the biotechnology revolution indicates that, in addition to fast and also incremental organizational change, the rapid multiplication of novel technologies nurtures numerous original business models, which constitute entirely new organizational forms. In addition, in biotechnology business model innovation is very much affected by governmental regulation. For instance, if governments put strict controls and barriers on the diffusion of biosimilar drugs, then the markets where these controls are enforced will require different business models than those where the controls are less rigid.

### Empirical evidence of an innovation cascade in biotechnology

Based on our conceptualization above, this section turns to empirical evidence of an innovation cascade in biotechnology.

Indicators of innovations abound, and the most evident is the spectacular rise of patenting (Kortum and Lerner, 1999; Dang et al., 2010). Patents are good indicators of invention, but not innovation, although there is often a link between invention and innovation. Many major innovations have started with a patented novelty. In the last decade, the number of US patents granted to assignees based in an increasing number of countries has grown very fast. In the United States and Canada it has doubled, in Israel it has more than tripled, and in China it has increased by a factor of 28.

Social science observers of the unfolding of biotechnology, since its irruption in the economic world in the 1970s, are divided about its importance. Some of them believe that biotechnology is a revolution in the making with large impacts on human and animal health, agriculture and even finance (Davis 1991; Zucker and Darby 1995; McKelvey 1996; Rifkin 1998; Moody 2004; Junkunc 2007). Other observers believe the biotechnology revolution is a myth, and that empirical evidence does not support the presence of a revolution, particularly in its human health applications (Nightingale and Martin 2004; Pisano 2006; Hopkins et al. 2007).

Different indicators can measure the knowledge explosion in the cascades: they include patents, publication, and products, for particularly scientific and technological knoweldge. Based on information from different parts of the new biotechnologies, Figs. 1, 2, 3, 4, 5, 6, 7, 8, 9, 10, and 11 give an indication of the fast expansion of new disciplines as measured by publication and patents.

**Fig. 1:**
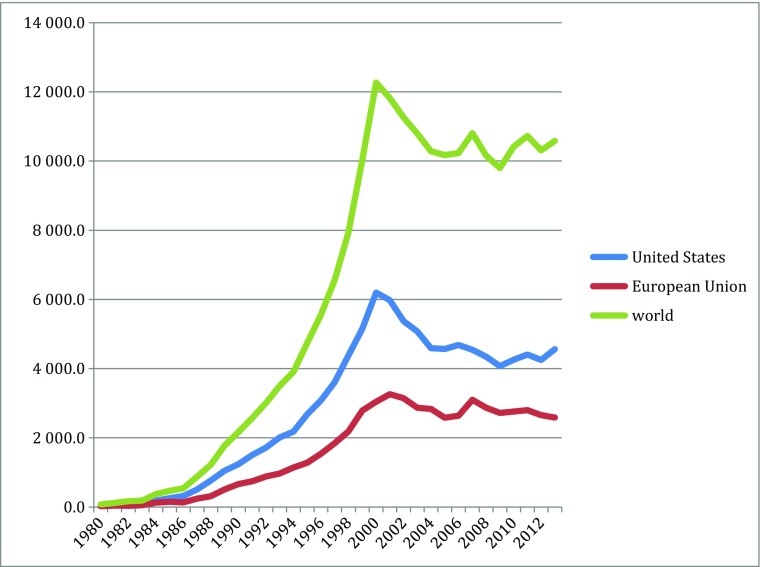
Triadic biotechnology patents (1980–2013): main assignee countries. Source; OECD

**Fig. 2:**
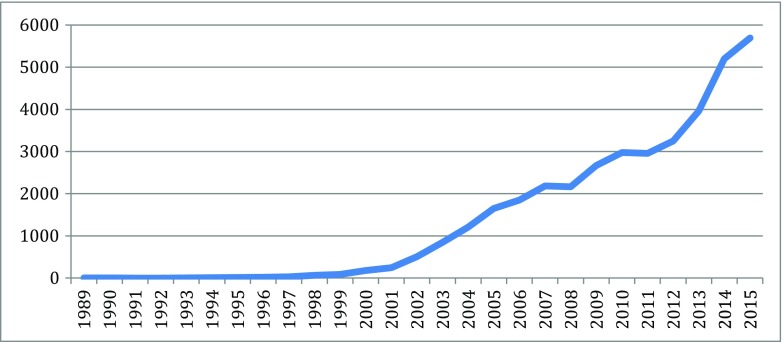
Bioinformatics articles (37,779 articles from 1980 to 2015). Source: SCOPUS

**Fig. 3:**
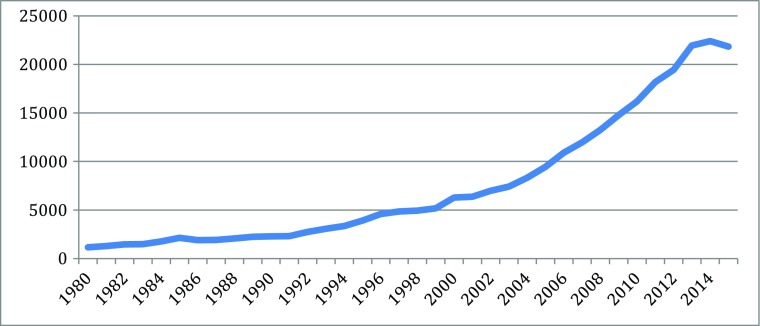
Stem cell articles (270,389 articles from 1980 to 2015). Source: SCOPUS

**Fig. 4:**
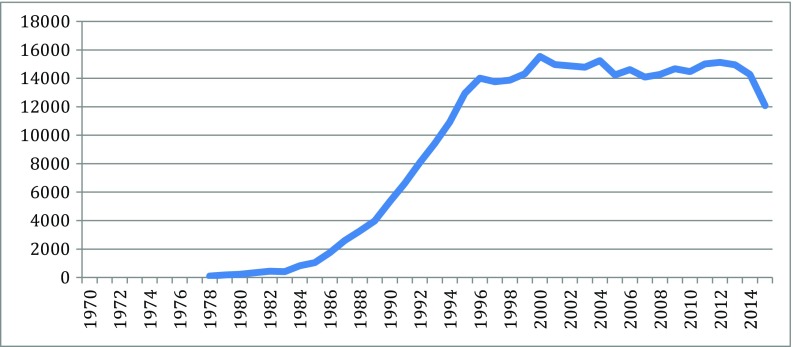
Protein articles 1978–2015. Source: SCOPUS

**Fig. 5:**
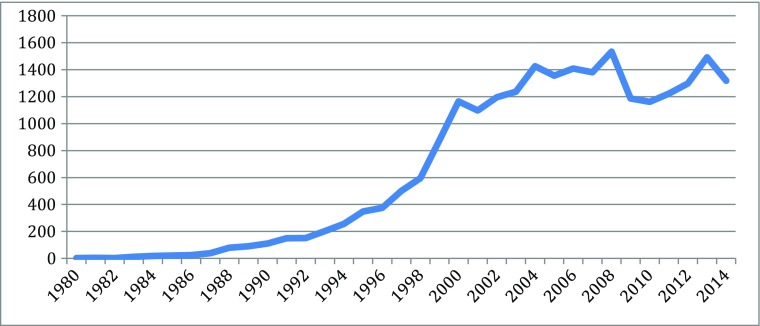
Recombinant protein patents

**Fig. 6:**
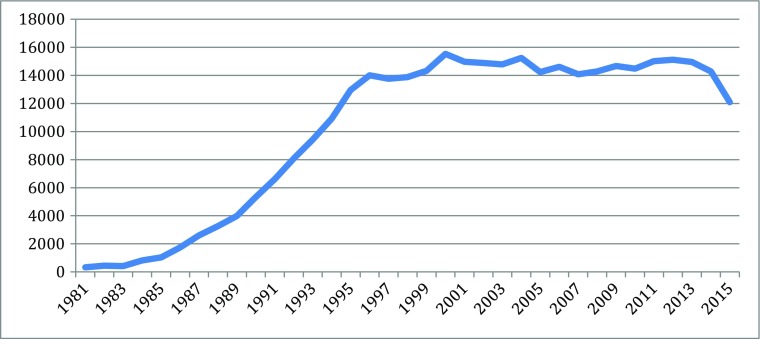
Gene therapy articles (160,054 from 1980 to 2015)

**Fig. 7:**
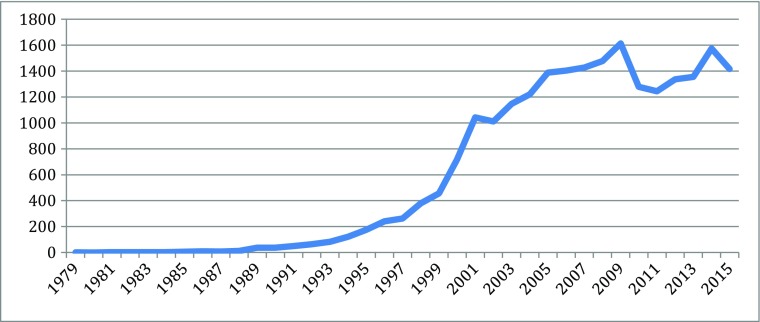
Gene Therapy PCT patents (23,645 between 1979 and 2015)

**Fig. 8:**
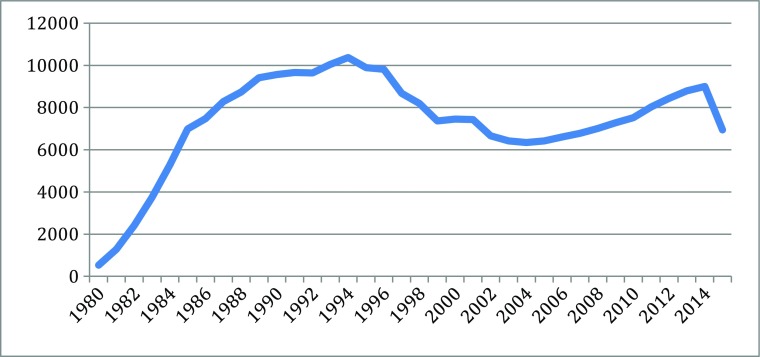
Monoclonal antibody articles 1980–2015

**Fig. 9:**
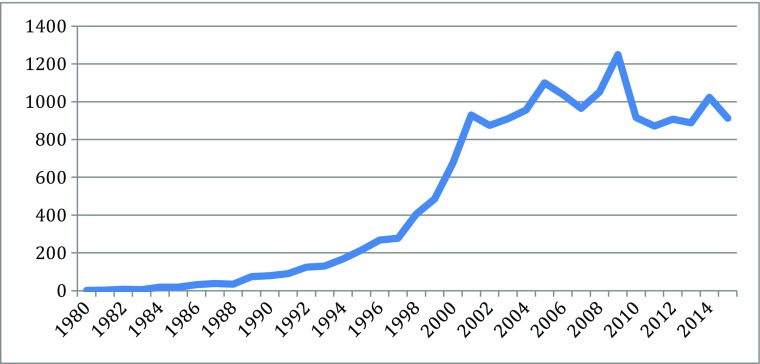
Monoclonal antibody PCT patents (1980–2015)

**Fig. 10:**
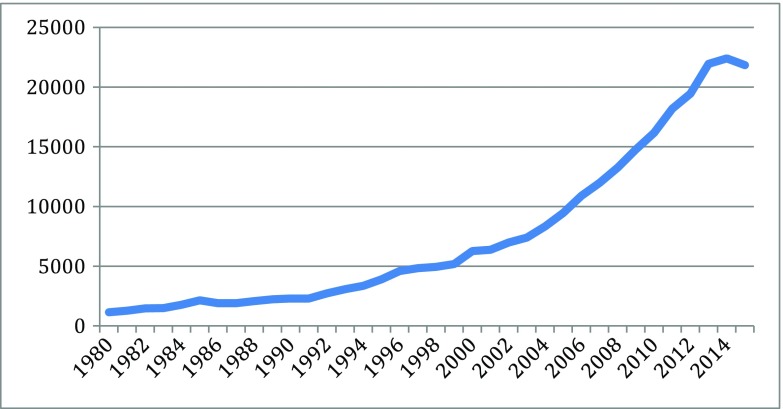
Stem cell articles, 1980–2015

**Fig. 11:**
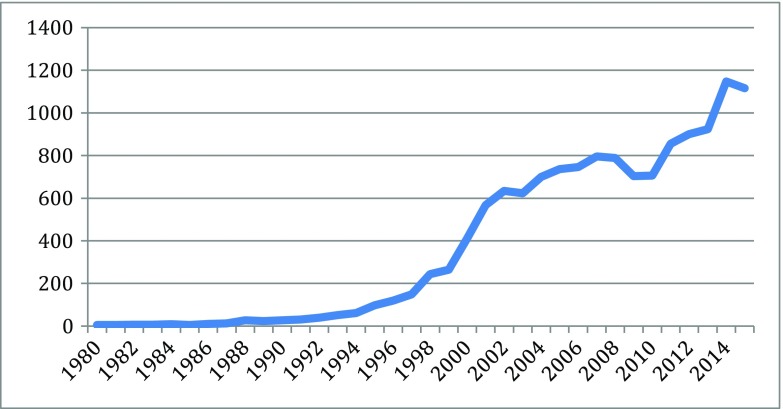
Stem cell PCT patents, 1980–2015

As indicated in the figures above, in the new biotechnology innovation cascade, the speed and rise of new knowledge is evident through the patent record. The rise of biotechnology scientific publication is even faster.

Measuring the institutional and organizational change offers less ready-made indicators. However, we argue that clearly we can identify key individuals and organizations involved, which have stimulated many new innovation cascades within biotechnology, as evidenced in Table 1.

**Table 1:** The biotechnology innovation cascade: some landmarks

Year	Discipline	Landmark event	Definition/goal	Key organizations and companies
1953	Biology	Drs. F. Crick and J. D. Watson (UK) discover the structure of DNA	Deoxyribonucleic acid (**DNA**) is a molecule that encodes the genetic instructions used in the development and functioning of all known living organisms	University of Cambridge, UK
1970	Bioinformatics	E. A. Kabat (USA) pioneer computer methods for biological sequence analysis	“The application of computer technology to the storage, management, and analysis of biological data.” (EBI, 2012, p. 4)^a^	Genomodel, Integromics, Rosetta, SymBioSys,
1972	Biotechnology: genetic engineering	Drs. H. Boyer and S. Cohen (USA) develop methods to combine and transplant genes	“Any technological application that uses biological systems, living organisms, or derivatives thereof, to make or modify products or processes for specific use.” (UN)^b^	Stanford University, Amgen, Biogen, Genentech, Gilead, Serono, Vertex
1975	Monoclonal antibodies (MABS)	Drs. C. Milstein and G. Kohler (UK) develop hybridoma techniques to produce MABS	The development of monospecific antibodies made by identical immune cells, cloned from a unique parent cell. They bind to any organic substance, that they can detect, purify or destroy	University of Cambridge, UK Abbott, Amgen, Biogen, Eli Lilly, Genentech Genzyme, Glaxo, Novartis,
1977	Genomics	Dr. F. Sanger (UK) publishes a key method to sequence DNA	Genomics is a discipline in genetics that applies recombinant DNA, DNA sequencing methods, and bioinformatics to sequence, assemble, and analyze the function and structure of genomes (the *complete* set of DNA within a single cell of an organism)	Agilent, Illumina, Life Technologies, Myriad Genetics, Pacific Biosciences, ^c^
1994	Proteomics	Dr. R. Nelson (USA) develops the use of mass spectrometry in immunoassays	The large scale study of proteins, their structure and functions	Applied Biomics, Biacore, Proteome Sciences…^d^
1998	Stem cell therapy	Drs. Thompson and Gearhart (USA) develop stem cells	Introduction of adult stem cell in damaged tissue in order to treat disease or injury, I.e. bone marrow transplantation	Mostly experimental in hospitals and research universities
2002	Gene therapy	Dr. Claudio Bordignon (Italy) publishes the first successful gene therapy treatment	Use of DNA as therapeutic agent to treat genetic diseases (replace mutated genes)	San Raffaele University, Italy, Ark Therapeutics Group, Ceregene (US) Glybera (Netherlands), Shenzen SiBiono GeneTech (China), Oxford BioMedica (UK)
2003	Genomics	Completion of the Human Genome Project (International)	The application of genomics concepts and technologies to the study of drug activity and metabolism, including gene expression, or inactivation and SNP association studies	AnyGenes, DeCode Genetics, Gentris, Glaxo, Jackson Library, ^e^
2003	Proteomics	Human Proteomics Atlas (Sweden)	A first atlas of the human proteome, required to understand the functions of the human proteins	The Royal Institute of Technology, Sweden
2010	Proteomics	Launching of the Human Proteome Project (International)	“The Human Proteome Project (HPP) is designed to map the entire human proteome in a systematic effort using currently available and emerging techniques.” (Legrain et al. 2011)	Academic and PRO institutions and research centres from some 20 nations

^a^European Bioinformatics Institute (EBI)(2012): In a Nutshell, Cambridge, UK

^b^The Convention on biological diversity. (http://www.cbd.int/convention/articles/default.shtml?a=cbd-02)

^c^ http://www.marketwatch.com/story/genomics-companies-ripe-for-flurry-of-mergers-2013-04-16

^d^ http://www.proteinscience.com/companies.html

^e^ http://www.jazdlifesciences.com/pharmatech/leaf/Drug-Discovery/Clinical-Research-Services/Pharmacogenomics.htm

These key events open up business model innovations for both existing firms as well as small entrepreneurial ones and for universities. As an illustration, note that the combination of actors may take advantage of smaller pilot projects (i.e. the Human Protein Atlas), but they thrive on grand-challenge policies such as the Human Proteome Project. Such large projects tend to create new paths more than they follow existing paths. The Human Genome Project, for example, has launched new paradigms in history, palaeontology, medicine, and a dozen other disciplines and technological paths, including forensic sciences.

In summary, our argument here is that not only biotechnology changing in radical ways, but that more interestingly, an innovation cascade can be observed, which is linked also to the resources of firms and to the intellectual capital of star scientists, as explained in the next section.

## The biotechnology innovation cascade and its determinants

Thus, based on the above evidence, we propose that an innovation cascade is visible in biotechnology. In doing so, we are proposing that innovation cascades are rare phenomena that require the combined impulse of several technological and institutional factors. Biotechnology is a good phenomenon to study using this lens, given the large number of potential new trajectories that have been opened up in recent decades. This section explores determinants in this case.

### The technological and scientific determinants

High-tech, science-based cascades need institutional support through knowledge producing organizations, such as research universities and public research organizations, but also venture capital industries and a legal and regulatory environment conducive to innovation. In the absence of such institutions, no high-tech science-based sector has ever seen the light. These institutions provide knowledge, but also highly skilled personnel, funds, markets and regulations.

Yet the trajectory of the cascade is difficult to predict (Niosi 2016). On the one hand, today there are many different research organizations in several dozen countries, within the OECD and among emerging countries, and all of them contribute to the cascade. Also, knowledge flows are easier than ever before from one country to the other and from one research institution to another. The number of scientific articles and patents in each of these high-tech industries is growing very fast, thus increasing the number of combinations of this new knowledge.

The biotech innovation cascade started in the 1950s with the discovery of the triple helix nature of DNA by Watson and Crick in the United Kingdom. The two main loci of the cascade became the United Kingdom (UK) and the United States (USA). In the 1950s, Cambridge professor Frederick Sanger was able to sequence insulin. In the USA, in the early 1970s, Elvin Kabat and his colleagues at Columbia University pioneered the computational study of genetic material. In California, Cohen and Boyer developed the technologies required to combine and transplant genes. A few years later, the first biotechnology company, Genentech, was founded (McKelvey 1996). In the meantime, Drs. Milstein and Kohler developed the methods necessary to produce monoclonal antibodies. The two decades that followed witnessed enormous development of the methods used to sequence genes. These developments paved the way to the Human Genome Project, an international endeavour that was concluded in 2003, with the sequencing of the entire human genome, and unleashed a new series of cascades, including pharmacogenomics, gene therapy and the Human Proteome Project (another multinational project) whose effects are now unfolding. In the meantime, stem cell therapy was developed in North America and several European countries, as well as in South East Asia. Gene therapy appeared in Europe, China and the United States.

Table 2 indicates the Pearson correlations for papers and patents, in different scientific fields underlying biotechnology.

**Table 2:** Pearson correlations between SCOPUS articles and PCT patents

Patents articles	Gene therapy	Stem cell	Recombinant protein	Monoclonal antibodies	Bioinformatics	Total PCT patents
Gene therapy	0.9	8158	14,680	12,631	1634	22,607
Stem cell	69,354	0.93	8318	6776	1298	13,549
Recombinant Proteins	53,350	28,829	0.94	13,079	1785	23,339
MAB	31,045	19,241	43,359	0.08	1379	17,741
Bioinformatics	1624	1298	1785	1042	0.8	2322
Total articles	159,981	270,389	357,692	264,570	37,792	79,338

### Internal to firm resource determinants

Not all biotechnology companies participate in the innovation cascade, and that is why one must bring the resource-based view into understanding innovation cascades. The innovating companies are those that have the adequate resources, mainly human resources, to accomplish the task. At least two main theoretical currents need to be recalled here, and in our view, both are necessary and must be integrated as two sides of this complex, emerging process of innovation cascades in biotechnology.

One of them is the resource-based perspective that started with Edith Penrose (1959), developed with the resource-based theory of the firm Wernerfelt (1984), expanded with Prahalad and Hamel (1990) competence theory and was extended by Teece et al. (1997) with the dynamic capabilities approach. Companies that are able to design new business models, implement them and modify them under the conditions of these cascades are those that have the appropriate managerial capabilities. They are also able to attract new human resource competences, and build up and implement new organizational structures, thus producing new business models.

The second current that needs to be recalled is the biotechnology intellectual capital that Lynn Zucker and her colleagues at the UCLA have pinpointed. Successful biotechnology companies are using advanced human capital (Zucker et al. 1998, 2002). Star scientists are transferring knowledge from universities to dedicated biotechnology firms (DBFs), through different mechanisms: founding new DBFs, acting as scientific counsellors of these firms, or working as scientific directors of their laboratories. In this way, they transfer fresh and yet tacit knowledge to the firms. It is to be noted that the intellectual capital current initiated by Zucker and her colleagues about biotechnology has not been linked to the more general resource-based theory of the firm, and its improvements in the competence and capabilities theory of the firm.

One contribution of this article is to underline the links between the approaches, as both aspects are required to get an innovation cascade moving within a science-based industry. In summary of our argument, in order to produce innovative business models, biotechnology companies need both competent managers and star scientists able to understand such a rapidly changing knowledge basis. Yet in order to produce not a single high-performing company, but a cascade, these capabilities must be common across many firms (Eisenhardt and Martin 2000). In our case, there must be numerous well-trained biotechnology managers and star scientists available. The United States is the country where this kind of human capital is most often found. It is not by chance that most components of the cascade are active in this country (see above, Table 1).

### Policy factors fuelling biotechnology innovation cascades

Additionally, such cascades require enormous financial resources to fund scientific and technological knowledge. Since the 1970s, the United States, the United Kingdom and other advanced nations have made human health biotechnology a priority. In the US, public investment has come through different channels. By 1984, the NIH spent 4.4 billion on R&D, out of which 1.5 billion was on applied drug R&D and $300–350 million on biotechnology (CBO 1985). The National Institutes of Health created in 1988 the National Center for Biotechnology Information (NCBI), which operates the GenBank, a nucleic acid sequence database. It also provides other key biological data. NCBI affords resources for both genomic and proteomics research. In 2015, its R&D budget for biotechnology is US$5.9 billion.^3^ This sum does not include cancer research, arthritis or other disorders where biotechnology may play an important role.

In addition, the United States played a key role in the Human Genome Project (HGP), through two funding agencies, the Department of Energy (DOE) and NIH. The HGP started in 1990 and was declared complete in 2003. The United Kingdom, Canada, France, Australia, China and Brazil were also partners in the project. The human genome was announced sequenced in 2003. It identified over 20,500 genes of *Homo sapiens*. The sequencing of the human genome created a revolution within the revolution: entire new disciplines emerged, such as genomics, pharmacogenomics and bio-archaeology. Other disciplines were transformed, such as forensic sciences and evolutionary biology. The HGP also started a revolution in innovation policy: it opened the era of “grand projects” in life sciences. The Human Protein Atlas was started in 2003 in Sweden, and is funded by the Wallenberg Foundation. Its goal is to study the entire human proteome. The first draft of the human proteome was published at the end of 2014 (Marx 2014).

In the UK, genomics has been a priority for decades. It started with Crick and Watson’s discovery of the double helix structure of DNA. It continued with Frederick Sanger, a biochemist (twice a Noble Prize in Chemistry) who first developed methods for sequencing proteins in the 1970s. Sanger worked at Cambridge University all his life. In 1993, the UK opened the Sanger Centre, today one of the largest genomics institutes in the world. After Sanger, the UK continued to invest in genomics and put this new discipline at the top of its Bioscience Technology Strategy (UK Biotechnology Strategy Board 2009). Such research has helped to discover the genetic basis of numerous diseases, as well as the interactions between genetic propensities and human behaviour and environment.

However, even smaller countries can help stimulate innovation cascades through pilot project, and thereby draw upon international alliances and resources. In 2003, the Swedish Royal Technology Institute (KTH) launched its Human Proteome Atlas Project, a mapping of the human proteome, the first draft of which was published in late 2014. The project was originally located at the KTH, but soon it recruited groups from other Swedish universities. In addition, formal collaborations took place with research teams in India, South Korea, Japan, China, Germany, France, Switzerland, USA, Canada, Denmark, Finland, The Netherlands, Spain and Italy.

In the meantime, in 2001, a much larger initiative in the area is underway, where twenty nations launched the Human Proteome Project (HPP). The Human Proteome Organization (HPO) coordinates it. In 2008, the cost of the HPP was estimated at US$ 1 billion (Pearson, 2008). Yet, according to some observers, the difficulty of the endeavour is such that the project will continue for many years in the future (Humphrey-Smith 2004). Its total cost will also increase over time. Its participants include organizations based in the United States, China, Australia, Brazil, Canada, France, Japan, India, Iran, Italy, the Netherlands, New Zealand, Russia, Singapore, South Korea, Spain, Switzerland, Sweden, Taiwan and Thailand. The results are expected to have a large impact on the development of diagnostic, prognostic, therapeutic and preventive medical applications. New business models will emerge from this new round of discoveries.

In sum, the governments of the most large and affluent OECD and emerging countries are investing yearly billions of dollars in national and international research projects developing the new life sciences and technologies. Through these big-science investments, they help fuel the innovation cascade.

### The biotech revolution and its business models

We agree with many observers in the human health, agriculture and industrial biotechnology, who consider that this new generic technology is at the basis of a major scientific, industrial and technological revolution (Hoffmann 1988–89; Kane 2012; MacGregor 2003; Juma 2011). Authors point out new drugs against previously unbeaten human diseases, such as AIDS, arthritis, cancer, haemophilia, hepatitis, herpes, influenza and rabies. Gene therapy and stem cell treatments are in their infancy but they are progressing in previously unimaginable ways and at remarkable speed. The production of food has multiplied, particularly in the main crops, due to genetically modified plants. Industrial biotechnology is being used in fermentation techniques and the separation of industrial minerals. In the area of animal health, numerous advances are registered. As Rifkin (1998) has put it: “The problem is that biotechnology has a distinct beginning but no clear end.” In other words, the biotech innovation cascade is here to stay.

Human health biotechnology products are also revolutionizing the pharmaceutical industry.^4^ In addition to the fact that six of the ten most sold drugs in the world are biotechnology products, the value of biopharmaceutical medicines has attained US$120 billion in 2013, and it grows at 6–8% a year. The best-selling drug, Humira was the top-selling drug in 2012 with a total of $9.3 billion. A third of all new medicines introduced worldwide are monoclonal antibodies (Mark 2013). Also, there are over 120 original biological drugs and dozens of biosimilars (similar drugs to original biopharmaceuticals) on the market. Diseases that seemed intractable 50 years ago are now being tamed. They include arthritis, cancer, and hepatitis. The 5-year survival rate of people diagnosed with cancer in the United States has increased from 49% in 1975–7 to 68% between 2002 and 2008 and keeps growing; the National Institute of Health attributes this important change to two factors: better medical imagery, and monoclonal antibodies and other biopharmaceutical drugs (American Cancer Society 2013). In addition, there are over 400 biotech drugs in clinical trials, targeting more than 200 different diseases.

Biotechnology is also at the origins of a cornucopia of business models. More specifically, a “business model” is a system of interconnected and interdependent activities that determines the way the company does business with its customers, partners, and vendors (Amit and Zott 2012). It defines the organization’s value proposition and its approach to value creation and value capture (Teece 2010).

This section organized the discussion of business models in biotechnology around one main synthetic taxonomy, and the text subsequently discusses evidence in references from specific countries, sectors and underlying scientific disciplines.

McKelvey (2004) identifies two existing dominant business models as well as ten emerging business models in life sciences. The taxonomy is based on the dichotomies of whether they primarily do activities in-house or through a network and whether they primarily exploit scientific or technological opportunities (McKelvey 2016). These ten new models, she argued, were emerging at the same time as the two dominant models of classical biotechnology model and vertically integrated model of pharmaceuticals were under high pressures to change.

The two existing models of biotechnology that McKelvey (2004) identifies are: The classical biotechnology model depends on long-term basic research, where they are specialized suppliers of knowledge, reliant upon positive externalities of knowledge from especially universities. However, given the disappointing past results, they have increasing difficulties to attract capital, given the risk and long-term nature of their research and product development. The vertically integrated model as pharmaceutical companies tried to hold all assets in house, including many complementary assets and product development. They have been challenged by the lack of new blockbuster drugs to support extensive internal R&D, as well as a shift towards open innovation to use collaboration instead. Given that these two business models are disintegrating under pressures to reform, there has been space to experiment with new models.

Thus, The biotechnology revolution created a new original business model that mimicked that of the large pharmaceutical corporations, called the *biotechnology blockbuster model* (McKelvey and Orsenigo 2006; Bradfield and El Sayed 2009). From 1960 to 2000, big pharmaceutical companies invested close to US$1 billion to develop each new chemical drug, submitted it to the Food and Drug Administration and other national health agencies, and protected it through a barrage of patents. In due time, those patents expired and generic companies entered the fray with generic drugs, less expensive but often lacking the quality control label of the large R&D pharmaceutical corporation. Two business models existed at that time.

The arrival of the new dedicated biotechnology firms (DBF) changed the structure of the industry. The DBFs tried to copy the blockbuster business model of big pharma, but only a few of them succeeded. Thus, new business models appeared. Some DBFs and big pharmaceutical firms decided to develop new drugs but only up to clinical essays Phase II, and then licence them to big pharma. This is called an “*out-licensing business model*”. Conversely, other companies prefer an “*in-licensing business model*”, where they purchase fairly advanced R&D projects from other companies, and guide them through the latest phases of clinical essays and eventually to the market (Schafer 2003). Other DBFs preferred to commit themselves only to the research phases, and often develop a *scientific services business model* in which they sell scientific research services to both big pharma and larger biotechnology firms. Still other firms became *specialized producers of biosimilars*, segmenting the market for different group of patients. Novo Nordisk, a Danish pharmaceutical giant, is a case in point. Other DBFs confine themselves to producing diagnostics tests. However, if the related drug is not approved, or if the diagnostics company makes an insufficient return on in its diagnostics kit, its investment may be lost.

Our taxonomy also proposes ten emerging business models, which are differentiated along two dimensions (McKelvey 2004). One dimension is a relative emphasis on either in-house competencies or else coordination across actors. The second dimension is a relative emphasis on whether the firm chooses to compete on technology or else compete on market and users. The five experimental business models where the firms compete primarily on technology (and therefore depend upon public research) include the platform model, the contract research model, the information model, the hybrid technology model, and the pure tool and component model. The two experimental business models where the firms compete primarily on market and customers (and therefore depend upon private and public health care providers and other firms) include the service-provider model and the market maker model. The three speculative business models (which may not exist but theoretically could) are the systems integrator model, the orchestrator model, and the open source model.

One can also examine specific areas, within this broader taxonomy (Table 3). Sha and de Noronha (2007) found a specific set of business models in China, which they categorize into: a) Reagent producers; b) Equipment and services; c) Generic drugs (biosimilars); d) Technology platforms (I. e. genetic analysis of fruit; bioinformatics software); e) R&D products/services (i.e. CROs); f) Hybrid of technology and products.

**Table 3:** Typology of emerging biotech business models

	Compete on in-house competencies	Compete on coordination across actors
Compete on market expertise	Service-provider Market maker	Orchestrator
Compete on scientific and technological experties	Platform model Contract Research Hybrid Technology	Information Pure tool and component Systems Integrator Open Source

Khilji et al. (2006) elaborated on different types. They found a *traditional business model* that sees biotechnology innovation as a long and expensive road leading to the discovery of new medicines (evident in the cases of Amgen, Biogen and Genentech). Over time, the dedicated biotech firm (DBF) becomes a fully integrated life science company. This sequential model needs venture capital, corporate venture funds and hundreds of millions of dollars. Few companies were able to fulfil the promise of this business model. This is a science and technology push model. This traditional model does not correspond to LDC economies. There is almost no VC, no institutional infrastructure and few star scientists. Moreiver, newer integrated business models require market knowledge on specific needs in particular markets. They depart from the original model**.** Multiple feedback loops linking funding, R&D, market institutions and internal functions of the biotech organization characterize them.

Varieties of these business models identiified in the taxonomy above have been found in different industiries using biotechnology and in different countries. Patzelt et al. (2008) find two major biotechnology business models in pharmaceuticals: 1) *Therapeutics firms* are dedicated to the development of bio-therapeutic drugs. The development of these products is expensive, time-consuming and risky, thus the business model may not progress too much among biotechnology firms. This model is close to the original blockbuster model.2) *Platform firms* either sell their technologies on the market or conduct classical fee-for-service business with customers. For example, they offer the application of their proprietary technology as a research service to other biotech firms and research institutes, which in turn pay for the service they have received. Nosella et al. (2005) found that different business models depend on the stages of the innovative process where they operate in Italian biotechnology; 1) Pharmaceutical companies working exclusively in the research market; 2) Integrated companies, which proceed from research to commercialization; 3) Companies which sell products to other biotechnology companies; 4) Firms that carry out industrial development in addition to manufacturing and commercialization; and Companies that produce and sell services.” (Nosella et al. 2005). The authors did not clarify whether these classes are mutually exclusive and comprehensive. A study of Indian biotech business models could confirm four different types found in the above taxonomy (Konde 2009). They could empirically identify: 1) *Platform*: under this model, companies develop a set of tools and use them to provide services, or sell them to users; the advantage of this model is that it provides faster revenues than the others. This model was used in genomics, bioinformatics and other niches; 2) *Product* model: that of firms developing drugs, either new to the world or new to the country; those developing “new-to-the-world” innovations usually run serious risks and often fail; 3) *Vertical* model: firms conduct R&D, clinical trials, and market their products like a pharmaceutical company; 4) *Hybrid* models combine platform and product strategies, and they sell both services and goods. Finally, some DBFs focus on orphan-drugs as their main product.” Orphan diseases are those that “manifest in population representing at the maximum 6-8% of the world population…” (Sharma et al. 2010). They include diseases for which there are only a few thousand patients, and those for which potential patients are so poor that they would not pay for the medicine, even when there are millions of them, such as the Ebola pandemic in Western Africa, and other tropical diseases. Incentives provided by the United States, Japan and the European Union governments help companies to reduce the cost of R&D and clinical essays of such orphan drugs. Biological drugs represent two thirds of the orphan drug market, one that had reached US$84.9 billion in 2009 and is supposed to reach US$112 billion by 2014 (Sharma et al. 2010).

Moreover, new business models are emerging, due to he arrival of bioinformatics, genomics, proteomics, gene therapy, stem cell medicine, related disciplines, and personalized medicine. These can be seen as a more specific varieties of the typology proposed above.

Bioinformatics introduced the possibility of personalized medicine. Bioinformatics also produced a plethora of business models, including those of companies collecting and licensing data, writing and licensing software and systems, selling products and services to perform testing, but also performing tests and creating stem cell databanks. Some of these technologies and business models can be patented (Gatto 2001; Fernandez et al. 2013).^5^ Authors find two main business models in bioinformatics: the more traditional *web-server models* where users interact with the pre-installed applications, a less flexible option; and the emerging *cloud-computing models* that allow on-demand allocation, of one of two varieties, a “resources-on-demand” model and a “pay-as-you-go” model. Both allow more personalized services to the client (El-Kalioby et al. 2012). Eagle Genomics, a Finnish bioinformatics company supported by TEKES, the Finnish national agency for research and development, offers the two alternatives: a web server service for a fixed price, and an “Eagle on demand” model for loosely defined and evolving projects (Holland 2013).

As to *genomics*, a recent study has identified five basic business models (Vanhala and Reijonsaari 2013): 1. Comprehensive genomics tests for consumers and as genome data bank material; 2. Genomics as part of individual health planning; 3. Services based on comprehensive genome tests; 4. Medical precision tests for consumers; 5. Restricted genetic trait tests. Most of these business models required a clinical office or at least a medical professional that monitored the genomics diagnostics.

*Pharmacogenomics produced another set of business models*. Some pharmaceutical companies, aware of the fact that most drugs are effective only on a limited set of patients, required the services of specialized pharmacogenomics firm in order to find out which patients would tend to respond favourably to the new drug. A paradigmatic case is the R&D and launch of Herceptin (trastuzumab), where pharmacogenomics services helped target the right patient segment for the new monoclonal antibody (Smart and Martin 2006). The fusion of genetic diagnostics and therapeutic research has produced another new term and business model: *theragnostics*. In this model, diagnostics companies and pharmaceutical or large dedicated therapeutic biotechnology firms work together to produce both the drug and the diagnostics; in another, the *independent model* under which the diagnostic company develops a diagnostics kit and related metrics, so that patients, the clinical organization, and/or large pharmaceutical companies pay the diagnostics company not on the basis of cost, but of value (Ferrara 2007). Thus, governmental health organizations, using very reliable diagnostics kits that allow fairly precise forecasts of the likely chances that a drug is effective for a group of patients, pay diagnostic companies on the basis of value: i.e. the number of patients [2 MOTS enlevés] healed [2 MOTS enlevés] after their identification by the diagnostic kit. Let us remember that today six of the ten best selling drugs are monoclonal antibodies. And the price of the new biological therapies is steep: in the United States, the average cost for the top nine biological drugs is more than $200,000 a year. Thus, any diagnostic kit based on pharmacogenomics that can accurately predict which segments of patients will be healed has huge value both for the patients, the insurance companies and government health organizations.

*Gene and stem cell therapies and their business models are still works in progress*, but large pharmaceutical corporations (such as Glaxo, Pfizer) and small firms (i.e. Novocell) are dedicating million of dollars to master the process of running such new segments of the biotechnology cascade. At least two new business models are now identifiable. They are: 1) *A repository model*: Some companies are offering repository services of umbilical cords for future uses of stem cells, for eventual autologous transplants of patients, or allogeneic transplants among compatible patients, usually family members. 2) *Cosmetics*: Other companies are using stem cells to launch new cosmetic products, based on stem cells, some of which are already on the market.

For investors, a major problem seems to be that both gene therapy and stem cell treatments are difficult to produce in other forms than in case by case individual cures, as is already done in the treatment of leukemia and in new cornea production. In Australia, some 1000 bone marrow transplants are carried out every year; most of them are autologous.^6^ In the United States, some 18,000 transplants of bone marrow or umbilical cord stem cells were performed in 2011; almost 60% were autologous, and another 17% among family members.^7^ Such figures illustrate the fact that stem cell therapies are still case by case individual treatments.

Finally, *personalized medicine business models* are now emerging. Pharmaceutical companies are still interested in the blockbuster drug that will increase sales by the billions. Yet, due to the increasing price of drugs and growing genomics knowledge, instead of administering such drugs to large populations on a trial-and-error basis, with large percentage of failure, governments request some estimation of the chances of success of these drugs. Companies offering individual profiles and diagnostics services are now emerging, in addition to those that produce those “omics” datasets, such as genomics, transcriptomics, epigenomics and metabolomics data, as well as data on clinical phenotypes and environmental factors (German Federal Ministry of Education and Research 2013).

In summary, the new biotechnology as innovation cascades has opened up a variety of new business models as identified above classified in the proposed taxonomy and with rich additional information. We consider these emerging business model innovations a necessary part of creating and diffusing intellectual capital which tests and uses a variety of specialized resources within the biotechnology innovation cascade.

## Conclusions

This article has proposed a conceptualization of innovation as a process, where the scientific and industrial application of technological knowledge nurtures new routines and institutions. In doing so, we have explained how business model innovations is related to innovation cascades. Moreover, we have used this conceptualization to illustrate a more sophisticated understanding of emerging business models and innovation cascades in biotechnology. We have shown how and why experimentation through these business models is possible through the accumulation of resources, as well as specialized knowledge.

Our analysis of innovation cascades in the case of biotechnology demonstrates the degree of radical change, in terms of scientific and technological knowledge as well as in institutions and firm routines embodied in business model innovations. Thus, we side clearly with social science observers who aregue biotechnology is revolutionary in many sectors (Davis 1991; Zucker and Darby 1995; McKelvey 1996; Rifkin 1998; Moody 2004; Junkunc 2007). We contend that observers which argue that the biotechnology revolution is a myth, particularly in its human health applications, have missed the point (Nightingale and Martin 2004; Pisano 2006; Hopkins et al. 2007). They seem to be still focused on genetic engineering and the blockbuster model of the 1970s and 1980s. Potentially, a reason for our findings as contradicting theirs is that we examine a much more complex sector, including many new branches and applications of biotechnology, and its rise to the top of the pharmaceutical industry. The cascades we found are both the result of innovation activities of large and small firms, not just large oligopolistic ones like in Mytelka and Delapierre (2003).

In terms of policy recommendations, grand challenge innovation policies are often – albeit not always – the best policy instrument that governments use to promote the cascades and channel the course of the process. In life sciences, several grand challenge policies have likely produced major results, like in genomics and proteomics. Institutional variables affect the number and viable models for biotechnology activities. Yet, the impetus of scientific and technological developments is such that they overwhelm institutional barriers and constraints, and provide raw material for new business models. Even so, some innovation systems seem to stimulate more experimentation and concentration of specialized knowledge resources. Hence, it may be possible that in those countries where public and private funds nurture the development of biotechnology, many innovative biotech business models will prosper also in the future.

For future research, interesting question address the role of policy and the appearance of innovation cascades and multiple business models in other sectors, especially renewable energy, information and communication technologies, and in nanotechnology. Our study is of course limited to biotechnology. However, we would like to propose that every rapid succession of technical changes brought about by multiple radical innovations will produce a similar cascade of business models. The resulting feedback process of rapid business model change and emergence may have an impact on technical change. In contrast, nothing of the sort appears to be occurring in renewable energies, where the cascade takes place in a more disordered way, despite significant investment in science policy, and hence a discussion of potential “policy failure” as well as “policy success”.

Another topic within the case of biotechnology is to provide further evidence and specification of the proposed taxonomy of emerging business models (McKelvey 2004) and of the role of developing countries (Niosi et al. 2012). Due to the rapid pace of technological change and the creation of entire new disciplines,^8^ biotechnology firms and large pharmaceutical companies are forced to experiment, design and assess new business models. Many varieties are outlined above could be further defined. A final topic for future research is to examine the interwoven nature of different innovation systems. Conversely to Antonelli (2008, 2009), our analysis indicates that innovation cascades are taking place in regional, national and global systems. Indeed, one proposition to explore from this paper is that science-based industries may more often national and global processes, even if their birth may be regional. Another is to focus upon co-evolution.

Finally, many research developments could interlink our conceptualization with different methodologies. We propose that interlinking business model innovations and innovation cascades may bring enormous promise to the innovation field. Indeed, even if several authors have mentioned system dynamics and complex adaptive systems, we propose more work on models need to be built of the different cascades that unfold today in the global economy. Agents need to be identified, knowledge flows scrutinized, networks analyzed. The world needs agent-based models to cope with rapidly changing and adapting learning systems (Bonabeau, 2002; Farmer and Foley, 2009). And, such models should be matched by qualitative and analytical studies of innovation cascades, in order to unravel the dynamics. The community may also find a plethora of agent-based models and system dynamics software able to show and analyze such fast-innovation periods.

## Footnotes

1 As of July 2016, only seven papers appear in SCOPUS with “innovation cascades” as keywords; only seventy-five were present on Google Scholar.

2 Lane, Maxwell and others do not make any statement about the size of firms launching the cascade. Conversely, Delapierre and Mytelka (2003) link innovation cascades to oligopolistic strategies.

3
http://report.nih.gov/categorical_spending.aspx

4 New biotechnology products in human health include synthetic hormones, new diagnostics, vaccines and many other products

5 As of October 20, 2015, a search in the USPTO database found 178 patents using the words “business model” in the abstract.

6
http://www.leukaemia.org.au/treatments/stem-cell-transplants/stem-cell-transplants

7
http://bloodcell.transplant.hrsa.gov/about/general_faqs/index.html#1990%20number%20tx%20in%20US

8 The latest one may be lifeomics, the merger of genomics, bioinformatics, proteomics, metabolomics, epigenomics and other brand new disciplines (He 2013).
